# Supplementary material for: An A. thaliana mutant lacking all nine ATG8 isoforms provides genetic evidence for functional specialization of ATG8 in plants
Source: J Cell Sci. 2025 Sep 10;138(17):jcs263803. doi: 10.1242/jcs.263803 (PMC12450472; doi:10.1242/jcs.263803)
Supplement: Supplementary information [file joces-138-263803-s1.pdf]

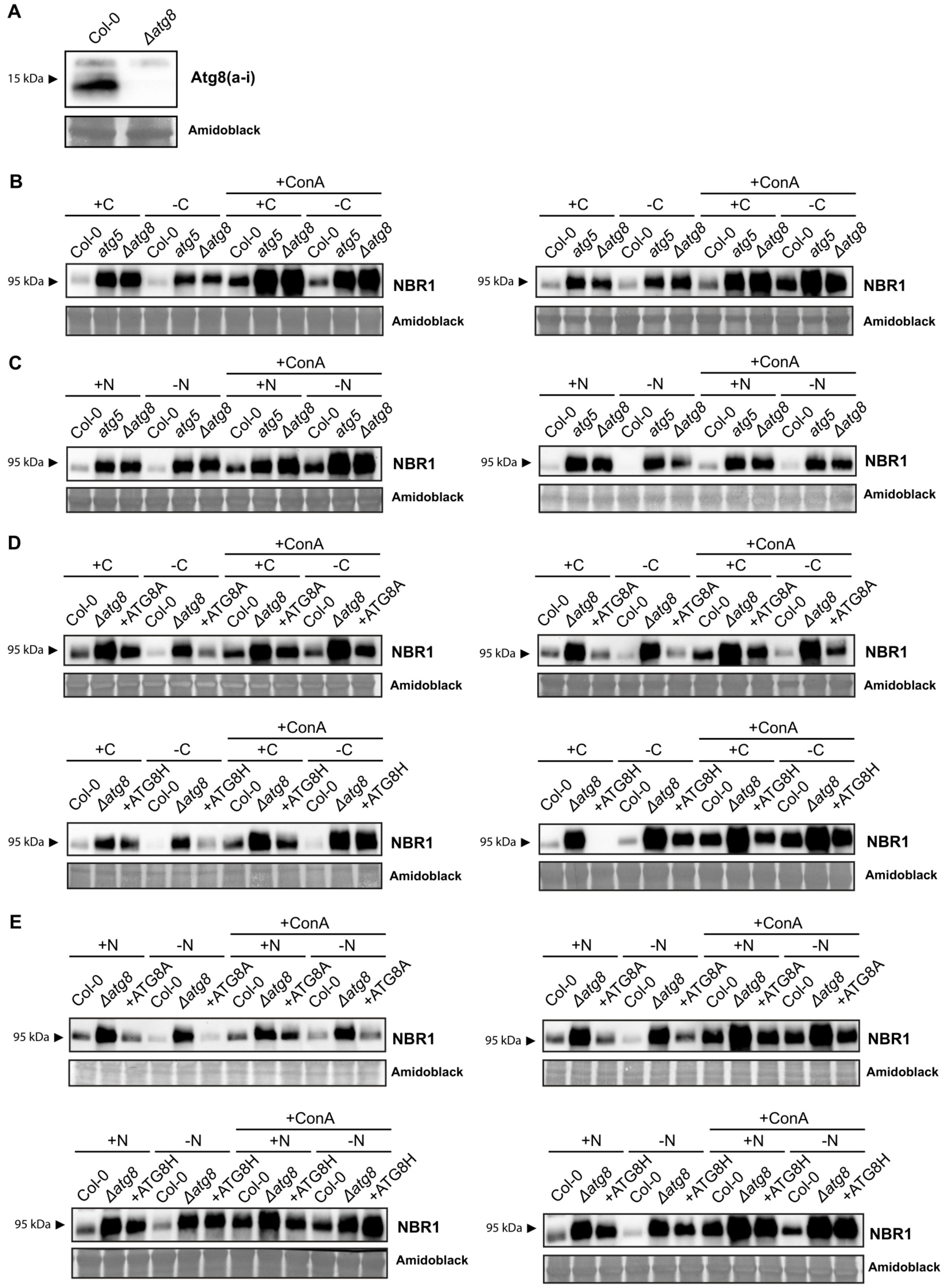

Fig. S1. (A) Western blot comparing endogenous ATG8 protein levels in Col-0 and Δatg8 mutant. (B-E) Replicates of western blots in Fig. 2 (D, F) and Fig. 4 (C, E, G, I).

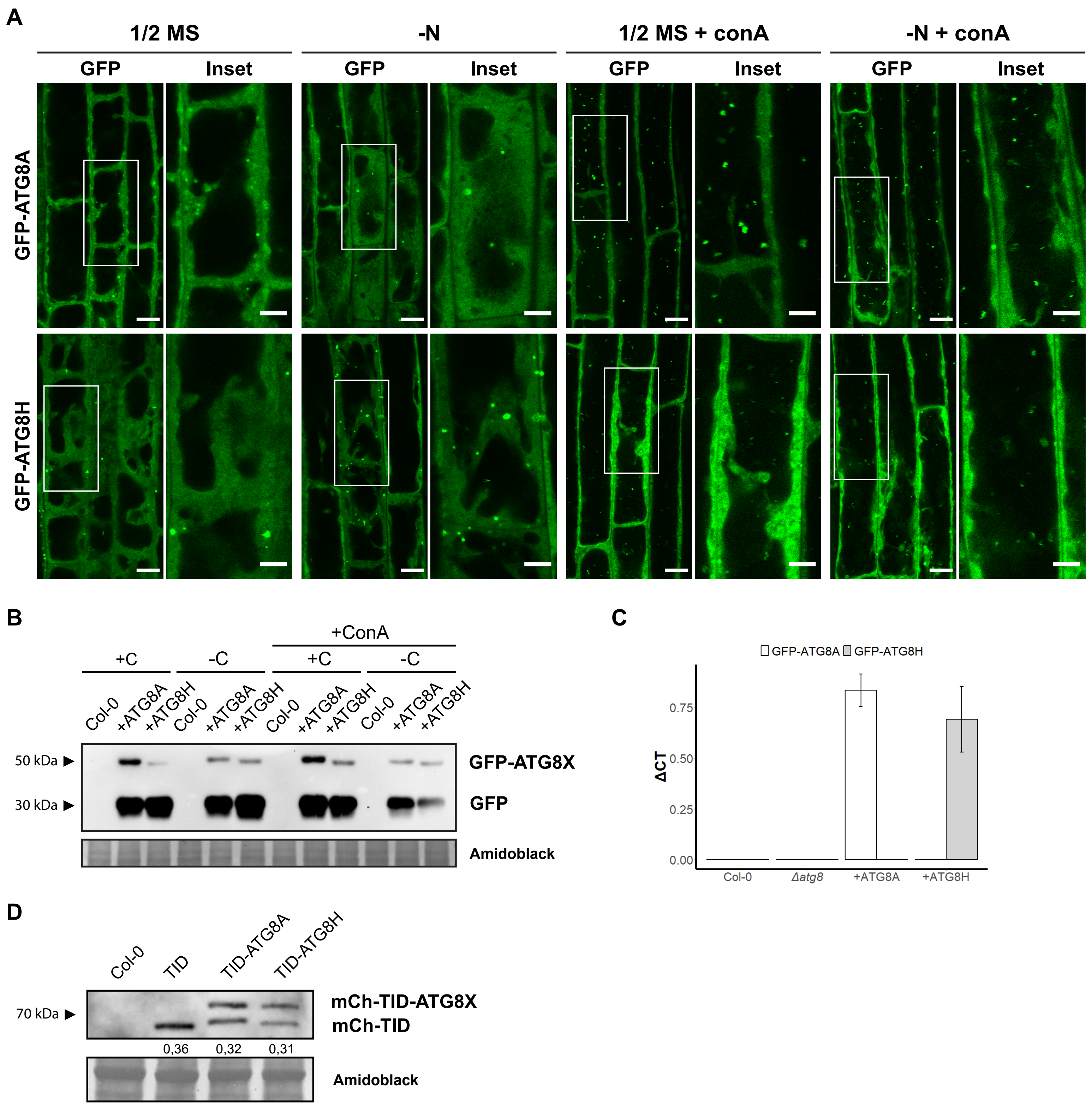
A
1/2 MS
-N
1/2 MS + conA
-N + conA
GFP
Inset
GFP-ATG8A
GFP-ATG8H
B
+ConA
+C
-C
Col-0
+ATG8A
+ATG8H
50 kDa
30 kDa
GFP-ATG8X
GFP
Amidoblack
C
GFP-ATG8A
GFP-ATG8H
ΔCT
0.75
0.50
0.25
0.00
Col-0
Δatg8
+ATG8A
+ATG8H
D
Col-0
TID
TID-ATG8A
TID-ATG8H
70 kDa
mCh-TID-ATG8X
mCh-TID
0,36
0,32
0,31
Amidoblack

Fig. S2. Arabidopsis ATG8 complementation lines have normal autophagosome structure and autophagic flux under control and nutrient-deficient conditions. (A) Representative confocal microscopic images showing the autophagosomes and the autophagic bodies inside the vacuole in root epidermal cells of the complementation lines Δatg8 /+GFP-ATG8A and Δatg8 /+GFP-ATG8A. 5-days old Arabidopsis seedlings were incubated in ½ MS liquid media or nitrogen-deficient (-N) liquid media for 3 h, or ½ MS liquid media or nitrogen-deficient (-N) liquid media containing 2 μM concanamycin A for 2.5 h before imaging. Representative images of 3 replicates were shown here. Scale bars, 10 μm. Inset scale bars, 5 μm. (B) Western blot comparing autophagic flux of complementation lines Δatg8 /+GFP-ATG8A and Δatg8 / +GFP-ATG8A upon C starvation treatment, in combination with concanamycin A (1μM). (C) Bar plot representing ΔCT values of GFP-ATG8A and GFP-ATG8H fusion genes from RT-qPCR analysis in Col-0, *Δatg8* and complementation lines Δatg8 /+GFP-ATG8A and Δatg8 /+GFP-ATG8A. Values were calculated as mean of three technical replicates and error bars represent the mean ± standard deviation (SD). (D) Western blot comparing expression levels of the fusion proteins mCh-TID, mCh-TID-ATG8A and mCh-TID-ATG8H. Relative quantification of protein bands is reported below the blot.

Journal of Cell Science • Supplementary information

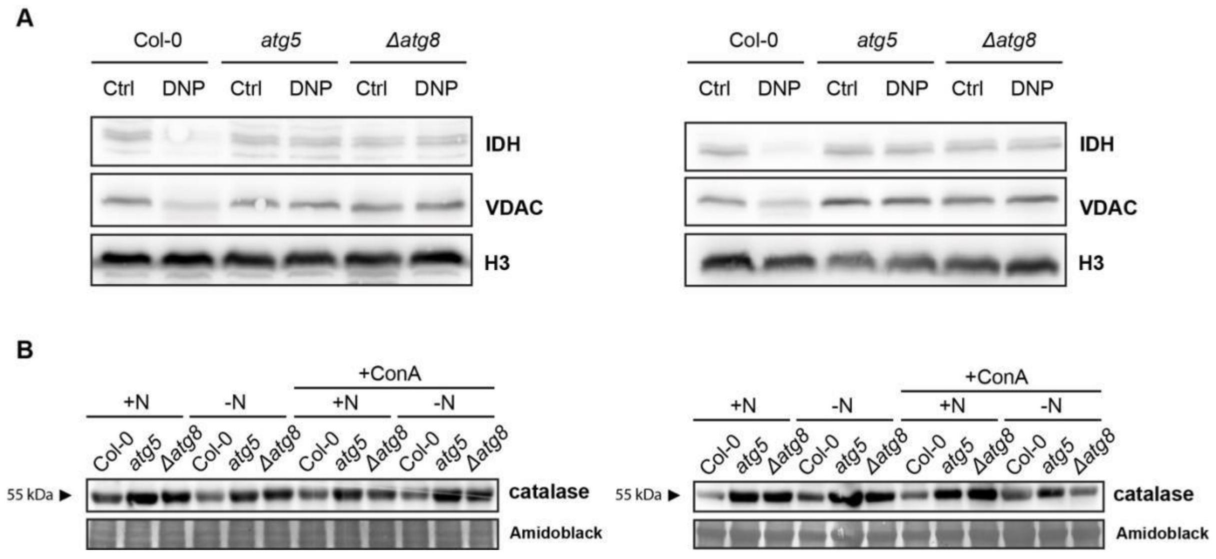

Fig. S3. Replicates of western blots in Fig. 3(A, D).

Table S1. Sequences of ATG8 proteins after CRISPR mutagenesis

Available for download at
https://journals.biologists.com/jcs/article-lookup/doi/10.1242/jcs.263803#supplementary-data

Table S2. Raw data for ATG8 proximity labeling proteomics

Available for download at
https://journals.biologists.com/jcs/article-lookup/doi/10.1242/jcs.263803#supplementary-data

Table S3. ATG8A and ATG8H proxitomes upon nitrogen starvation

Available for download at
https://journals.biologists.com/jcs/article-lookup/doi/10.1242/jcs.263803#supplementary-data
